# An EPA database on the effects of engineered nanomaterials-NaKnowBase

**DOI:** 10.1038/s41597-021-01098-0

**Published:** 2022-01-20

**Authors:** William K. Boyes, Bradley Beach, Gayle Chan, B. Lila M. Thornton, Paul Harten, Holly M. Mortensen

**Affiliations:** 1 Center for Public Health and Environmental Assessment, Office of Research and Development, U.S. Environmental Protection Agency, 109 T.W. Alexander Drive, Research Triangle Park, Durham, NC 27709 USA; 2 Oak Ridge Institute for Science and Education (ORISE) appointee at Office of Research and Development, US Environmental Protection Agency Research Triangle Park, Durham, NC 27709 USA; 3 Evans School of Public Policy and Governance, University of Washington, Seattle, WA USA; 4 Department of Civil & Environmental Engineering, Duke University, Durham, NC 27708 USA; 5 Center for Computational Toxicology and Exposure, Office of Research and Development, U.S. Environmental Protection Agency, 26 West Martin Luther King Drive, MS 483, Cincinnati, Ohio 45268 USA

**Keywords:** Computational toxicology, Genetic databases

## Abstract

The US EPA Office of Research and Development (ORD) has conducted a research program assessing potential risks of emerging materials and technologies, including engineered nanomaterials (ENM). As a component of that program, a nanomaterial knowledge base, termed “NaKnowBase”, was developed containing the results of published ORD research relevant to the potential environmental and biological actions of ENM. The experimental data address issues such as ENM release into the environment; fate, transport and transformations in environmental media; exposure to ecological species or humans; and the potential for effects on those species. The database captures information on the physicochemical properties of ENM tested, assays performed and their parameters, and the results obtained. NaKnowBase (NKB) is a relational SQL database, and may be queried either with SQL code or through a user-friendly web interface. Filtered results may be output in spreadsheet format for subsequent user-defined analyses. Potential uses of the data might include input to quantitative structure-activity relationships (QSAR), meta-analyses, or other investigative approaches.

## Abstract (summary)

**Table:** 

Measurement(s)	engineered nanomaterial effects
Technology Type(s)	digital curation
Factor Type(s)	physicochemical property
Sample Characteristic - Environment	nanomaterial

Machine-accessible metadata file describing the reported data: 10.6084/m9.figshare.17060120

## Background & Summary

The recent advances of nanotechnology have led to concerns for the potential release of engineered nanomaterials (ENM) into the environment causing exposure to, and perhaps adverse effects on, humans or sensitive ecological species^1^. Accordingly, the United States Environmental Protection Agency (US EPA) Office of Research and Development (ORD) has developed a research program aimed at understanding the potential environmental implications of ENM. ORD research encompasses potential releases of ENM from manufacturing and commercial uses; environmental transformations, fate, and transport; exposures; and potential adverse health effects. A framework was developed to organize and integrate this diverse set of information^2^. To support this larger effort, a relational database was developed containing ORD nanomaterial research data to better enable the use and synthesis of study results, and to facilitate higher-order analyses such as quantitative structure-activity relationships (QSAR). One goal is to probe the relationships between physical and chemical properties of ENM and their environmental actions to see if predictive relationships can be determined. This publication announces the release of “NaKnowBase” (NKB), a knowledge base containing the results of multiple ORD publications on the actions of ENM in environmental or biological media.

The design of NKB was intended to compliment efforts in nanoinformatics – the strategic curation and collation of nanomaterial data for analytic purposes. A roadmap for nanoinformatics in the European Union (EU) and US was recently published providing a comprehensive overview of the inter-related scientific disciplines of nanomaterials science, physicochemical characterization, computational modelling, informatics, and ecological and human toxicology^3^. This analysis identified three challenges facing nanoinformatics: (1) limited datasets, (2) limited data access, and (3) regulatory requirements for validating and accepting computational models. NKB partially addresses the first two of these issues by providing a publicly available source of curated data relevant to ENM environmental health and safety (EHS). Collating datasets from multiple sources facilitates more comprehensive meta-analyses, QSAR, and risk assessment approaches such as read-across^4^. To date, such “big data” endeavours in ENM EHS tend to be designed around large datasets that must be generated in advance, or remain limited by a paucity of relevant, curated data from disparate sources^4–6^. Efforts like NKB can help overcome these research hurdles by being strategically designed to leverage extant data while also being amenable to newly generated data.

There are other nanomaterial-related databases indexed in the appendix section of the EU-US roadmap^3^. These databases are independently operated and vary according to the intended use and operability, the types of data captured, and the data format, access, and control. Although it may appear advantageous to consolidate these, there are several factors favouring the maintenance of independent databases: ability to control access to, quality of, and integrity of the data, managing and protecting proprietary and confidential business information, the pragmatics of scale, and the availability and continuity of funding. Therefore, the original scope of the NKB was limited to data collected by the EPA ORD. To our knowledge, the data provided in NKB are not collected elsewhere. The data in NKB represent the only collated source of published data from the US Environmental Protection Agency in a relational database regarding the potential environmental effects of engineered nanomaterials.

**Fig. 1:**
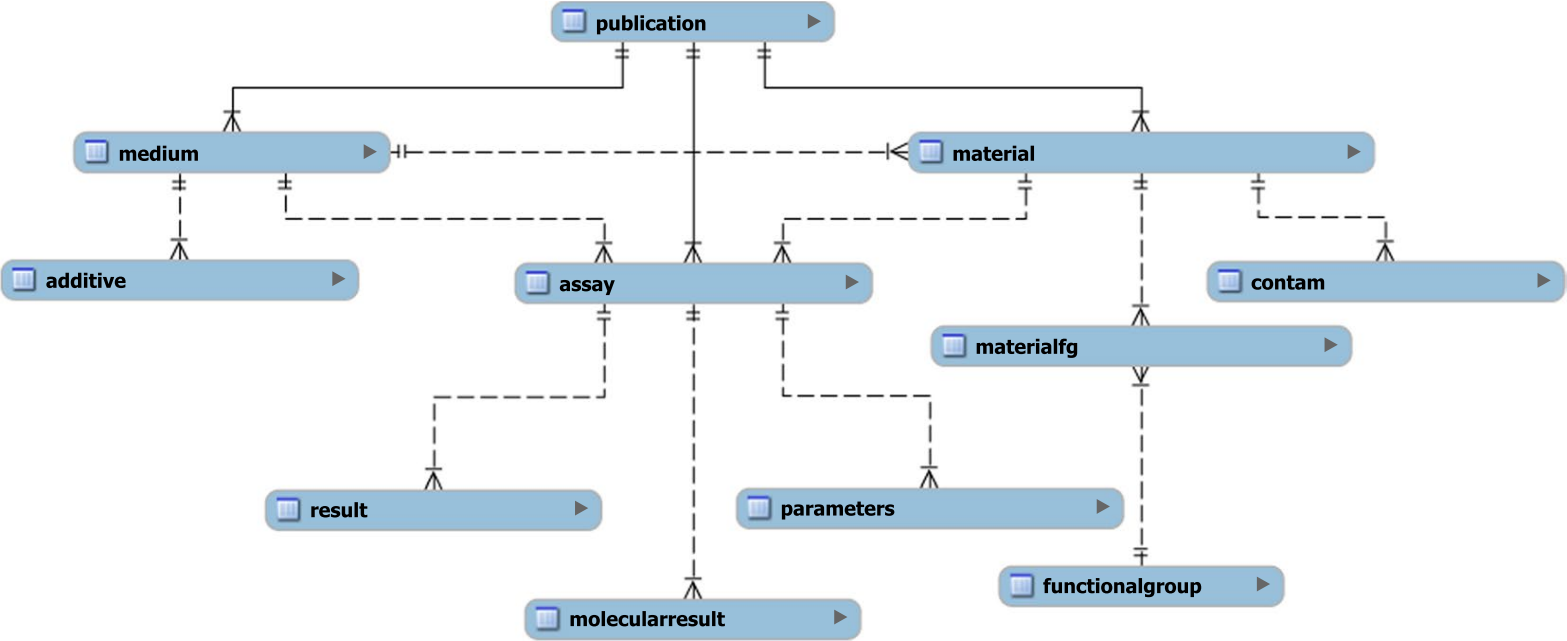
Overview of the NKB SQL structure. The lines indicate the nature of each relationship. Each relationship is of a one-to-many nature, where the end with two lines is “one” and the end with a triangle is “many”, such as one publication being able to have many mediums.

NKB was built as an SQL relational database. The overall structure is shown in Fig. 1. The database has separate tables on the source publication, the tested materials and their physicochemical properties, the media in which the materials were tested, the assays performed, the parameters evaluated, and the results. There are sub-tables to capture data on chemical contaminants, attached functional groups, and test media additives. Data entry is accomplished by curators via a set of prescribed Excel spreadsheets that are then imported to the database using a script. During curation, efforts are maintained to use terminology consistent with an expanded nanomaterial ontology being developed by several nanoinformatics groups including the EU NanoSafety Cluster and the Center for the Environmental Implications of Nanotechnology (CEINT), in coordination with the foundational work published by the eNanoMapper database^7,8^. In addition, a simple, user-friendly interface was developed which allows users to search the database and obtain outputs of data in spreadsheet format.

## Methods

Publications selected for curation were limited to research conducted by ORD and related to environmental or biological actions of ENM. This included *in vivo*, *in vitro*, and *in silico* experiments as well as life-cycle analyses and physicochemical characterisations. The data in the database reflect over 120 relevant publications from approximately 2012 through November 2019. Over 70 unique nanomaterials as defined by the combined composition of the core, shell and coatings were studied. Over 160 named assays and 22,000 individual assays were run. We expect to maintain the database and continue to make additions over time as new research becomes available. Though NKB will be made available through the Office of Science Management as a public EPA database tool, pertinent NKB data will also be integrated with the CompTox Chemicals Dashboard (https://comptox-prod.epa.gov/dashboard/chemical_lists/), which maps the DSSTox substance records to the most current list of NKB nanomaterials. The addition of new data will be announced via the CompTox Chemical Dashboard (https://comptox.epa.gov/dashboard/) on the ‘News’ (https://comptox.epa.gov/dashboard/news_info) and ‘Downloads’ (https://comptox.epa.gov/dashboard/downloads) pages of the Dashboard, as appropriate.

The EPA maintains various repositories for planned, ongoing, and completed research and projects. These repositories were searched for relevant publications for curation. The description and content of these repositories are detailed below.

### STICS

The Scientific & Technical Information Clearance System (STICS) is used by ORD to electronically approve and monitor scientific and technical products produced by ORD. STICS allows approved users with an EPA account and password (such as EPA employees and contractors) to search entries and download the results.

### Science inventory

The Science Inventory (SI) stores publicly available records about research conducted by the EPA, allowing EPA account-holding users to search through entries. Much of the database-relevant information in SI overlaps with STICS.

### Science hub

Science Hub is a data storage site for datasets associated with recently published EPA journal articles (beginning in 2016). EPA employees and contractors may access these datasets directly through Science Hub while the general public is granted access through a separate portal (The Environmental Dataset Gateway; https://edg.epa.gov/metadata/catalog/main/home.page).

### Direct input from investigators

Where available, ORD researchers provided their publication(s) and original data for inclusion in the database. These papers and submitted data were evaluated on a case-by-case basis and formatted by trained curators for inclusion in the database. Approximately 9% of the entries were submitted directly by the investigators. Among the reasons that original data may not have been available included the primary investigators having left the Agency, data having been archived, lack of access to raw data from scientific instruments, and incompatible formats. An example of an incompatible format was lists of differentially expressed genes encoded as “increased” or “decreased” where the data fields in the NKB required numeric value entries.

### Systematic article selection

Papers of interest were identified by running keyword searches through STICS, Science Inventory and Science Hub. A list of entries containing “nano” in the keywords or title were obtained. Additional queries were run separately using search terms including the composition of common ENM (e.g. silver, copper, titanium dioxide, cerium dioxide, etc.). Results were checked for duplicates, and posters, abstracts, or meeting presentations were not considered for curation. Over 600 titles were identified for further screening. These results were then reviewed to identify only original, peer-reviewed research. Finally, titles and abstracts were carefully read for relevance to nanotoxicology, environmental effects of nanomaterials, physical and chemical properties, and ENM life cycle. Other nanomaterial papers including literature reviews and those relating to topics such as incidental or naturally occurring nanomaterials, method development or “green chemistry” synthesis of nanomaterials were excluded.

### Table organization and curation procedures

The curation of data into the database required a set of trained data curators and a substantial commitment of time and effort. Artificial intelligence or other automated procedures were not used. The original training of data curators was generously conducted by the database experts of the Center for Environmental Implications of Nanotechnology (CEINT) in association with the Nano Informations Common (CEINT NIC), a database maintained at Duke University in Durham NC. Experienced NKB curators subsequently oversaw the training of new data curators as needed. Training consisted of explaining the overall purpose and structure of the database and the data input templates, and then overseeing the curation of selected model datasets which had been curated previously by others. When the novice curators were sufficiently proficient at capturing data from the training sets, they began with oversight to encode new manuscripts. Curators typically became proficient in a matter of a few weeks. Once curators were proficient, curation of data from each new manuscript typically required between one to several workdays depending on the complexity of the material. Questions or uncertainty about experimental procedures or parameters were referred to the project management and occasionally required contact with the authors of the original manuscripts for clarification. Thus, the robust curation of data for the database required considerable time and effort of skilled personnel.

Data extraction and curation occurred in accordance with an approved EPA quality assurance project plan (QAPP E-TAB-0030177, Project ID “Emerging Materials Project 18.02”). In summary, all data were collected from published journal articles. Metadata were attached to all curated data. Data were extracted from manuscript figures using a web application called WebPlotDigitizer (https://automeris.io/WebPlotDigitizer/). Modifications to curated data (for correction of curation errors, etc.) were logged and described in a separate text file.

Publications were added to NKB by entering metadata, experimental procedures, and results into a data collection template comprised of 11 preformatted Excel spreadsheets. Once completed, automated uploading of curation tables into database was accomplished by an in house Java program that transformed the contents of the templates into database-ready tables (csv files).

### SQL structure

The overall SQL structure of NKB is presented in Fig. 1, and a brief description of each data table is provided in Table 1. An overview of the fields and columns, in each NKB data table is further detailed in Tables 2–11. Field names are PascalCase to distinguish them from lowercase data table names. Primary keys, or fields comprised of unique identifiers for each entry in a data table, are listed first. Most tables use a single field as the primary key; the Material, Assay, and Medium tables use two keys. Primary keys and foreign keys are used to connect related data that are stored in different tables.

**Table 1:** An overview of the data tables in NKB, with a brief description of the general type or category of data collated in each table.

	Description
publication	Identification and metadata of the published manuscript from which the data originated
medium	The medium in which the nanomaterial was tested (e.g. water, saline, cell culture medium, etc.)
additive	Any substances that may have been added to the media (e.g. FBS, strep/pen)
material	The composition of the nanomaterial and any physiochemical parameters reported
contam	Any contaminants of the test material reported
materialfg	Functional groups affixed to primary test material and the method of affixation
functionalgroup	Identities of functional groups in database (e.g. alcohol groups)
assay	The type of test system used (e.g. *in vitro* test system, electron microscopy)
parameters	Parameters manipulated in the experiment (e.g. dose/concentration tested, test time, etc.)
result	Measured results linked to the assays and parameters employed
molecularresult	Pointer to results from complex assays such as genomics, proteomics, etc., that are deposited elsewhere

**Table 2:** Publication table data fields.

	Description
DOI	Unique Digital Object Identifier
PubTitle	Title of the publication
Year	Year of the publication
Journal	Journal of the publication
Volume	Volume of the journal
Issue	Issue of the journal
PageStart	Starting page number
PageEnd	Ending page number
Keywords	Keywords provided in the publication
Abstract	Publication Abstract
FirstAuthor	First and last name of first author; Middle name/initial included if included in publication author list.
Correspondence	Name of the author the paper indicates as handling correspondence.
Affiliation	Institutional affiliation of the author listed in Correspondence.

Curation of the data began with extracting the metadata and storing it in the publication table. DOIs were used as a unique identifier for publications. Additional metadata included publication title, journal title, volume and issue numbers, page numbers, publication year, abstract, keywords, first author, the point of contact, and the affiliation of the point of contact.

**Table 3:** Medium table data fields.

	Description
MediumID	Unique (within publication) numerical identifier to link a medium to entries in other tables
publication_DOI	Reference to DOI of source publication
MediumDescription	Name of test medium (e.g. water, saline, etc.)

Data regarding the dispersion mediums and any additives to the mediums were recorded. The Medium and Additive tables were used to track information on any medium a nanomaterial was suspended in during an experiment. Where multiple instances of media changes or particle characterizations were made over time, these were recorded in association with experimental time variables with appropriately linked experimental parameters. Mediums were uniquely identified by a combination of their source publication’s DOI and an incrementing number, MediumID, since one research publication could have studied multiple mediums. Complete medium data included the unique identification key and a description of the medium, such as a common name or a majority component (e.g. Dulbecco’s Modified Eagle Medium or water).

**Table 4:** Additive table data fields.

	Description
**AdditiveID**	Unique (within publication) numerical identifier to link an additive to entries in other tables
**Additive**	Name of additive
**Concentration**	Concentration of additive
**Units**	Units of additive
**medium_MediumID**	Reference to Medium ID of the medium this additive was added to
**medium_publication_DOI**	Reference to DOI of medium’s source publication

Additives to a medium were recorded in the Additive table. Entries in this table were comprised of the DOI and MediumID of the medium in question, the name of the substance being added, the amount being added, and the units. A medium could have any number of additives, including zero.

**Table 5:** Material table data fields.

	Description
MaterialID	Unique identification code for the tested material
publication_DOI	Reference to DOI of source publication
CoreComposition	Primary composition of the tested material
ShellComposition	Primary composition of a shell applied to the core substance
CoatingComposition	Primary composition of material applied as a coating to the core substance
SynthesisMethod	How the ENM was made: “Original method” if original, DOI of publication if a method is cited from a publication, or name of method if a common name is used.
SynthesisDate	When the ENM was made
CASRN	CAS Registry Number of core composition
Supplier	Source of the material
ProductNumber	Manufacturer’s product number
LotNumber	Production lot number
**ValueApproxSymbol/ Unit/Uncertainty/Low/High/Method**	
• OuterDiameter	Seven separate fields capture summary measurement information for each of the nine ENM characteristics in the bulleted list, totalling 63 fields. The field “OuterDiameterValue” is used for non-nanotube particle size measurements. ApproxSymbol captures characters used to qualify measurements that lack precision, typically due to limitations of the instrumentation used for measurement (e.g. <, >, ~). Low and High are defined by Uncertainty. If Uncertainty describes a concept with two numbers (e.g. range), Low and High hold the endpoints. If Uncertainty requires a single value (*e.g*. standard deviation), the value is stored in Low and High is left blank.
• InnerDiameter	
• Length	
• Thickness	
• SurfaceArea	
• SizeDistribution	
• Purity	
• HydrodynamicDiameter	
• SurfaceCharge	
Shape	The shape of the original particle
medium_MediumID	Reference to Medium ID of the medium this material was examined in
medium_publication_DOI	Reference to DOI of source publication
ShapeInMedium	Particle shape in identified medium
Solubility	Particle solubility in medium

Each entry in the material table was uniquely identified by the DOI of the source publication and an incrementing number to account for publications that studied multiple materials. Fields in this table address ENM composition, metadata (i.e., manufacturing information), and other physicochemical properties including, but not limited to, those addressed on EPA forms for submission of novel nanomaterials for registration under the Toxic Substances Control Act (TSCA) (https://www.regulations.gov/document?D=EPA-HQ-OPPT-2009-0686-0015). Note that companies were not required to generate data for these fields in order to submit TCSA registrations, only to report such data if available.

Core Composition was defined as the base material of the ENM, and any additions to the structure were recorded in Shell Composition or Coating Composition. Synthesis Method refers to a common method name or the DOI for a publication available. Core Composition was defined as the base material of the ENM, and any additions to containing the methodology. Several fields associated with large-batch or industrial scale ENM manufacturing are included: Synthesis Date, Supplier, Product Number, Lot Number, and if applicable, the Chemical Abstracts Service Registry Number (CASRN). Shape recorded the typical shape of the material, which was important for materials like carbon that varied wildly (*e.g*. sheets, tubes, or a simple bulk form). If the material was suspended in a medium, that medium was referenced by DOI and Medium. This allowed for important rows about medium-specific qualities, such as Shape in Medium or Solubility, to be captured. Specifically, NKB captures many quantitative characteristics for a nanomaterial, *e.g*. outer diameter, inner diameter, length, thickness, surface area, size distribution, purity, hydrodynamic diameter, and surface charge. Many publications report these data using summary statistics without raw data. Therefore, each ENM characteristic was described using a set of seven fields capable of capturing raw and processed data: Value ApproxSymbol, Unit, Uncertainty, Low, High, and Method. Average contained either the raw or average numeric value reported for a measurement. ApproxSymbol captured any qualifying characters (e.g. <, >, ~) denoting measurements that lacked precision, typically due to a limitation of the machine used for measurement. Unit contained the physical unit for the measurement, using standard scientific abbreviations when possible. Raw data were reported using these first three fields along with Method. The Uncertainty, Low, and High fields are used in combination to describe the spread or distribution of processed data. The Uncertainty field held statistical terms such as “range” or “standard deviation”. If the term required two endpoints, Low and High held the numeric values for those respective endpoints. For example, the Low and High of an “interquartile range” would be the first and third quartile values, respectively. If the “Uncertainty” statistic term required only one value (e.g. standard deviation), the value was recorded in Low. Finally, the technique or method used to produce the raw or processed measurements was recorded in Method (e.g., transmission electron microscopy).

**Table 6:** Contam table data fields.

	Description
ContamID	Unique identifier for the contaminant data point.
material_MaterialID	Reference to Material ID of the material in which this contaminant was found
material_publication_DOI	Reference to DOI of source publication
Contaminant	Chemical identity of the contaminant
ContamAmount	Measured numerical amount of the contaminant
ContamUnit	Units of measurement of contaminant (e.g. %, units of mass per volume)
ContamMethod	Analytical method to identify and measure the contaminant (e.g. ICP-MS, etc.)

The contaminants table, “contam”, served as an addendum to the material table. The primary key was comprised of the publication DOI and MaterialID of the contaminated material. The field Contaminant listed the name of the contaminating substance. ContamAmount, ContamUnit, and ContamMethod held the information on the scale of the contaminant and the way the contamination was measured. This allowed for a material to have any number of contaminants, each detailed in its own row.

**Table 7:** Materialfg table data fields.

	Description
MaterialFGID	Unique identifier for the functional group-material link.
material_MaterialID	Material ID of the material which has a functional group attached
material_publication_DOI	Reference to DOI of source publication
functionalgroup_FunctionalGroup	Chemical identity of the functional group.
FunctionalizationProtocol	Technical method to functionalize the material (e.g. acid wash, etc.)

Materialfg connects specific functional group data to the broader material data. If a material had functional groups, these were tracked in the functional group and materialfg tables. Functional group was a simple list of predefined functional groups. Each row in the materialfg table was a combination of a functional group, a material ID, a publication DOI, and the name of the functionalization protocol used to add the functional group to the material. A material could have any number of functional groups.

**Table 8:** Assay table data fields.

	Description
AssayID	Unique (within publication) numerical identifier to link an assay to entries in other tables
publication_DOI	Reference to DOI of source publication
AssayType	Type of assay performed (e.g. *in vivo*, *in vitro*)
AssayName	Name of the Assay performed (e.g. cell viability
medium_MediumID	Reference to Medium ID of the medium used in this assay
medium_publication_DOI	Reference to DOI of medium’s source publication
material_MaterialID	Reference to Material ID of the material used in this assay
material_publication_DOI	Reference to DOI of material’s source publication

The experiments performed in the publication were recorded in the Assay and Parameter tables. An assay was considered to be the experiment at large, while parameters were the experimental constants (such as the species being studied) and variables (dosage concentrations or exposure durations). Rows in Assay were uniquely identified through the DOI and an incrementing ID. Assays were assigned an Assay Type from a defined list of terms like “*in vitro*” and “*in vivo*”. AssayName held the common name for the experiment being performed. Each row in the assay table referenced a material and medium by their respective DOI-ID combinations.

**Table 9:** Parameters table data fields.

	Description
	Unique (within publication) numerical identifier to link a parameter to entries in other tables
ParameterName	Parameter evaluated in the assay (e.g. dose, time, etc.)
ParameterNumberValue	Numerical value of the parameter. Mutually exclusive with ParameterNonNumberValue.
ParameterNonNumberValue	Non-numerical value of the parameter. Mutually exclusive with ParameterNumberValue (e.g. natural light, a species, etc.).
ParameterUnit	Unit of value (e.g. percent, millimolar)
assay_AssayID	Reference to Assay ID of the assay this parameter helps define
assay_publication_DOI	Reference to DOI of assay’s source publication

Each assay was defined by one or more parameters, which were each stored in a row of the parameters table. All rows in the parameters table referenced an Assay by DOI and ID. Other fields included: ParameterName, ParameterNumberValue, ParameterNonNumberValue, and ParameterUnit. All parameters had a name but were restricted to either a numeric value and unit or a non-numeric value.

**Table 10:** Results table data fields.

	Description
ResultID	Unique (within publication) numerical identifier for the result data
ResultType	Type of results reported (e.g. viability)
ResultDetails	Any optional notes about the result
ResultValue	Numeric value of the reported result
ResultApproxSymbol	Used to note when a measurement is above or below the physical detection limits of the methods or machinery used (e.g. >, <, =)
ResultUnit	The units of the reported value
ResultUncertainty	States what uncertainty type is reported with the value, such as standard deviation or a range.
ResultLow	Holds the values described by Result Uncertainty. For ranges, this field holds the lower endpoint. If the uncertainty only reports one value (such as standard deviation), this field holds that value.
ResultHigh	Holds the upper endpoint for values described by Result Uncertainty. Is left blank for uncertainties with only one value reported.
assay_AssayID	Reference to the Assay ID of the assay this result came from.
assay_publication_DOI	Reference to DOI of the assay’s source publication.

This table was used to record the results of an assay. Each row in Results referenced an assay by DOI and ID. Since an assay could have multiple results, each row in Results was given an incrementing ID to serve as the primary key. The ResultName field specified what kind of result, or endpoint, was being reported (e.g. size, pH, mortality, LD50, etc.). ResultDetails included any additional information that ResultName could not capture. Finally, the seven fields used to capture raw and processed measurement data from the material table were used here to describe the result measurement or assessment.

**Table 11:** Molecularresults table data fields.

	Description
MolecularResultID	Unique (within publication) numerical identifier for the molecular result data.
assay_AssayID	Reference to the Assay ID of the assay this molecular result came from.
assay_publication_DOI	Reference to DOI of the assay’s source publication.
GEOAccession	Number used to access molecular result set in the NCBI Gene Expression Omnibus.
OrganismName	Scientific species name for the subject species.
SpeciesID	Unique identifier for the subject species on the NCBI Taxonomy Browser
AssayType	Details on the style of assay used to collect the data
Platform	Array, probe set, etc used to perform the assay.
Series	Reference number for the assay series.
SampleCount	Number of samples included in the results.
URL	Web address of the reported dataset.

This table was an alternative to Results used to store references to results that exceeded the capacity of NKB for complexity such as genomic, proteomic or metabolomic assays. Such results were typically already deposited on outside data repositories. NKB, in these cases, provided the ENM specific aspects and experimental design considerations of these studies, which could be linked to the large datasets housed elsewhere. Rows in this table catalogued web addresses to external sources for the results in question.

### NKB User interface

The NKB user interface application is currently under development. Deployment is expected in 2023 under the EPA web domain naknowbase.epa.gov. Here, curated data can be accessed through a user-friendly interface and search results can be downloaded for subsequent analysis by the user. NKB data can be filtered by numerous parameters such as ENM composition, physical and chemical characteristics, assay name and type, assay parameters, and result name. NKB data points are also linked to the original peer-reviewed publications via a single hyperlink.

The NKB user interface allows users to search for data using a pre-defined list of relevant search terms categorized by data tables and table fields. The searchable data fields were derived from those listed in Tables 2–11.

## Data Records

Figure 1 and Table 1 describe all the individual data sources integrated in NKB. The NKB data frame has been uploaded into a single collection entitled “NaKnowBase-SQL backend-080121” ^9^. The files contained in this collection include the most recent SQL data structure for NKB, including all tables, as well as corresponding data categories and keys for the backend of the database.

EPA nanomaterials present in NKB are also provided through the CompTox Chemicals Dashboard (https://comptox.epa.gov/dashboard/chemical-lists/NAKNOWBASE), which maps EPA chemical substance records to the most current list of NKB nanomaterial substance records (last updated 12/14/2020).

## Technical Validation

In general, there are many varied methodologies for cataloguing nanomaterials metadata and physicochemical properties; NKB attempts to capture as much of this information as possible.

Publications considered for curation were limited to ORD research, which is subject to rigorous internal and external quality control and peer review. All research conducted at ORD must have a corresponding Quality Assurance Project Plan (QAPP). QAPPs describe the necessary quality assurance and quality control measures needed to produce results that meet stated performance criteria. ORD OAPPs are peer-reviewed, approved by management, overseen by a quality assurance manager, and subject to periodic QA and performance quality checks. Manuscripts submitted for publication are linked to approved QA plans and are subject to QA review and approval. Furthermore, manuscripts are subject to thorough internal scientific peer review before undergoing additional external, independent peer review by the publishing journal. These systems are intended to ensure the quality and accuracy of ORD data, and help assure the reliability of data being curated in NKB. Because of this, the results of the papers themselves were not checked for errors during data curation. Instead, quality control efforts focused on ensuring the accuracy of the curated data compared to the original raw data, as well as consistent curation procedure between curators.

To assess the quality of NKB curation, a random sampling (approx. 5%) of curated papers were manually checked for quality control. It was found that data derived from the digitization of published graphs differed from the original data by an average of 0.20% ± 0.29% (N = 316) and that curation of the same data by different curators differed by an average of 0.33% ± 3.3% (N = 736). The data are calculated as Mean ± SD normalized to the axis scale.

## Usage Notes

Potential uses of the data include input to quantitative structure-activity relationships (QSAR), meta-analyses, or other modeling or investigative approaches. Users should be aware that data obtained from the NKB includes a large number of potential parameters related to physicochemical properties of ENM. Because relatively few of these properties were entirely consistent across sources, the NKB contains many sparsely populated fields. Users should consider this when planning analyses of data from the NKB. Updates to the NKB described herein help inform new testable hypotheses about the etiology and mechanisms underlying ENM effects in the environment and adverse health outcomes of toxicological concern in relation to human exposure to nanomaterials.

## Data Availability

All custom code created to process of manipulate external datasets in the construction or subsequent update of the NaKnowBase relational database tables are made publicly available by the U.S. Environmental Protection Agency, Office of Research and Development (ORD)^9,10^.
